# Local but Not Systemic Administration of Uridine Prevents Development of Antigen-Induced Arthritis

**DOI:** 10.1371/journal.pone.0141863

**Published:** 2015-10-29

**Authors:** Sudeep Chenna Narendra, Jaya Prakash Chalise, Mattias Magnusson, Srinivas Uppugunduri

**Affiliations:** 1 Autoimmunity & Immune Regulation (AIR), Department of Clinical & Experimental Medicine, Linköping University, Linköping, Sweden; 2 Department of Clinical Chemistry and Department of Clinical and Experimental Medicine, Linköping University, Linköping, Sweden; Queen Mary University of London, UNITED KINGDOM

## Abstract

**Objective:**

Uridine has earlier been show to down modulate inflammation in models of lung inflammation. The aim of this study was to evaluate the anti-inflammatory effect of uridine in arthritis.

**Methods:**

Arthritis was induced by intra-articular injection of mBSA in the knee of NMRI mice pre-immunized with mBSA. Uridine was either administered locally by direct injection into the knee joint or systemically. Systemic treatment included repeated injections or implantation of a pellet continuously releasing uridine during the entire experimental procedure. Anti-mBSA specific immune responses were determined by ELISA and cell proliferation and serum cytokine levels were determined by Luminex. Immunohistochemistry was used to identify cells, study expression of cytokines and adhesion molecules in the joint.

**Results:**

Local administration of 25–100 mg/kg uridine at the time of arthritis onset clearly prevented development of joint inflammation. In contrast, systemic administration of uridine (max 1.5 mg uridine per day) did not prevent development of arthritis. Protection against arthritis by local administration of uridine did not affect the anti-mBSA specific immune response and did not prevent the rise in serum levels of pro-inflammatory cytokines associated with the triggering of arthritis. In contrast, local uridine treatment efficiently inhibited synovial expression of ICAM-1 and CD18, local cytokine production and recruitment of leukocytes to the synovium.

**Conclusion:**

Local, but not systemic administration of uridine efficiently prevented development of antigen-induced arthritis. The protective effect did not involve alteration of systemic immunity to mBSA but clearly involved inhibition of synovial expression of adhesion molecules, decreased TNF and IL-6 production and prevention of leukocyte extravasation. Further, uridine is a small, inexpensive molecule and may thus be a new therapeutic option to treat joint inflammation in RA.

## Introduction

Rheumatoid arthritis (RA) is a chronic inflammatory, autoimmune disorder, predominantly affecting women. RA is characterised by enhanced leukocyte recruitment to the synovium, hyperplasia of the synovium and release of inflammatory mediators leading to pronounced joint inflammation and damage [1].

In RA patients, increased expression of the adhesion molecules ICAM-1, VCAM-1and E-selectin has been implicated in leukocyte adherence and susbsequent accumulation in the synovium [2]. Elevated levels of TNF, IL-1 and IL-6 in RA patients [3] promote increased expression of these adhesion molecules on synovium and leukocytes [2], leading to increased infiltration of leukocytes. Pro-inflammatory factors secreted by recruited cells activate and stimulate proliferation of resident synoviocytes, resulting in hyperplasia of the synovial lining layer—a characteristic hallmark of RA. Enzymes secreted by recruited neutrophils destroy cartilage and osteoclasts of the proliferating synovial lining layer are further activated to promote bone degradation [4]. Taken together, the increased extravasation of inflammatory cells to the synovium results in swelling and ultimately destruction of joints.

Currently, corticosteriods and methotrexate and are being used in combination as the first line of treatment for RA patients. Novel biological agents, mostly monoclonal antibodies, directed against TNF and IL-6 are being used for refractory disease and are efficacious in controlling inflammation in RA. However, long term use of these drugs has been hampered by serious adverse effects including resurgence of dormant malignancy, opportunistic infections and development of tolerance [5, 6]. Although several new therapies are being tested such as anti-CD19, anti-CD20, anti-IL-17 and anti-CD52 [7], there is still a need to explore other therapeutic options.

Recently uridine, a small inexpensive molecule has received attention for its anti-inflammatory properties in disease models of asthma and dry eyes [8–10]. We therefore evaluate its potential as an anti-arthritic therapy using antigen induced arthritis (AIA) which is an experimental model of Rheumatoid arthritis (RA), to study effect of uridine administration on arthritis development. In this model, arthritis is induced after intra-articular injection of the antigen in pre-sensitized mice, which results in an inflammation resembling RA in terms of synovial membrane hyperplasia, leukocyte infiltration and pannus formation [11–14]. Here we demonstrate that uridine can also protect against (antigen-induced) arthritis in a dose dependent manner, and we show for the first time that this protection is characterised by a dramatic suppression of synovial ICAM-1, CD18 expression and local expression of pro-inflammatory cytokines. We thus show that decreased synovial endothelial adhesion molecules expression is associated with hampered synovial leukocyte influx.

## Materials and Methods

### Animals

Healthy female NMRI mice (B & K Universal AB) aged between 8–10 weeks, weighing 30–35 g were kept at standard conditions of temperature and light, fed standard food and water ad libitum with 4 mice per cage in individually ventilated cages (IVC). Experiments were conducted according to the Swedish Welfare Act and approved by the ethical committee of Linköping University (Dnr. 66–10).

### Induction of arthritis

Briefly, animals were first pre-immunized subcutaneously with PBS containing 200 μg of mBSA and Freunds complete adjuvant (total 200μl) on day 0. Subsequently, animals were booster immunized with PBS containing 100 μg of mBSA and Freunds incomplete adjuvant (total 100μl) on day 7. Arthritis was induced by intra-articular injection of 30 μg of mBSA in 20 μl of PBS in left knee on day 21 in pre-immunized animals [15]. The experiment was terminated one week after intra-articular injection of mBSA (day 28). For euthansia, mice were injected with ketamine and medetomidine hydrochloride (intraperitoneal), after which blood was drawn by puncturing axillary vessels. Then mice were sacrificed by cervical dislocation. For each independent experiment we aimed to include at least 6–7 mice to allow reliable statiscal comparison between groups.

### Treatment protocol

The systemic effects of uridine on arthritis development were tested by systemic administration of 0–100 mg/kg of uridine. Uridine was injected subcutaneously on day 0, 7, 21 and 23 and intra-peritoneally (day 14) during AIA. In another series of experiments uridine was also administered by surgical insertion (subcutaneous in the dorsal neck region) of sustained release pellets releasing 0.75 or 1.5 mg uridine per day (Innovative Research of America, Sarasota, USA). For surgical insertion of pellets, mice were put under isoflurane anesthesia (inhalation) for the whole procedure. We investigated the potential anti-inflammatory effects of uridine when administered locally in the knee joint. Uridine 0–100 mg/kg was co-administered intra-articularly in the right knee joint together with mBSA (in 20 μl of PBS) on the day of arthritis induction (day 21).

## Histopathology and Immunohistochemistry

Knee joints were isolated on day 28 for histopathological and IHC staining. Tissue sections were prepared and stained with haematoxylin and eosin for arthritis evaluation as described earlier [15]. A double blind evaluation of arthritis severity was conducted by two separate observers. Specimens were judged on an arbitrary scale from 0–3 based on signs of inflammation defined as infiltration of cells in synovial tissue and synovial membrane thickening. Grade 0 denoted no signs of inflammation and grades 1–3 denoted increasing signs of inflammation.

Tissue sections were deparaffinised with xylene, rehydrated with decreasing strengths of alcohol and followed by washing in de-ionised water for IHC. Antigens were retrieved by heating tissue sections in sodium citrate buffer (pH = 6.0) for 20 minutes in a microwave oven. Sections were incubated with 0.3% Triton for 10 minutes and blocked with goat serum for 20 minutes followed by washing with PBS-Tween 20. Sections were then incubated with primary rabbit antibodies directed against murine TNF (ab9739-ABCAM, Cambridge, UK), IL-6 (BS-0379R- Bioss Inc., Massachusetts, USA), CD3 (BS-4815R- Bioss Inc.), F4/80 (sc-26643-R- Santa Cruz Biotechnology, Inc., Dallas, USA), neutrophil elastase (ab68672-ABCAM), ICAM-1 (BS-0608R- Bioss Inc.), CD18 (LFA-1) (BS-0503R- Bioss Inc.) and isotype control rabbit IgG (bs-0295p-Bioss Inc.). Bound primary antibodies were detected using IHC detection kit (ab64261-ABCAM) and DAB development was done according to the manufacturer’s protocol. Finally, all sections were counterstained with Mayers haematoxylin (Histolab products AB, Göteborg, Sweden). For Immunohistochemistry scoring of the sections was performed using an arbitrary scale from 0–5 based on intensity and extensiveness in a double blinded manner [16, 17]. A score of 0 represented no expression and 5 represented abundant expression.

### Determination of anti-mBSA IgG

ELISA plates (96 well, flat bottomed, Thermoscientific NUNC) were coated with mBSA (10μg/ml) diluted in 50mM carbonate/bicarbonate buffer and incubated overnight at 4°C and blocked by addition of 2% casein (Sigma-Aldrich, Stockholm, Sweden) for 2 hrs at RT. Samples diluted 1:500 in casein buffer were added in triplicate to the plates and incubated for 2 hrs at RT. Horseradish peroxidase (HRP)-conjugated secondary antibody goat anti-mouse IgG (Southern Biotech, Birmingham, AL, USA) diluted 1:4000 was added to plates and incubated for 2 hrs at RT. Plates were washed 3 times with 0.05% PBS-Tween 20 between each step. The plates were then developed by adding substrate, tetra 3,3′,5,5′-tetramethylbenzidine (TMB) (Sigma) followed by incubation in dark for 20 minutes. Finally, this reaction was stopped by adding 1 M Sulphuric acid and colour developed was quantified at 450 nm.

### *Ex vivo* assessment of antigen-specific proliferation

Spleens and draining lymph nodes were isolated at the end of the experiment. Single cell suspensions were prepared by crushing and passing spleens and lymph nodes through 70 μm nylon cell strainer. Red blood cells were lysed using red cell lysis buffer (Sigma-Aldrich). 2 x 10^6^ cells were seeded per well in a 96 well plate using Iscoves complete medium (4 mM glutamine, 50 μM β-mercaptoethanol, 100 U/ml penicillin, and 0.1 mg/ml streptomycin (Sigma-Aldrich) and re-stimulated with 50 μg/ml of mBSA for 48 hours. ^3^H-thymidine was added and cells were incubated for an additional 20 hours. Cells were harvested and the amount of incorporated tritiated thymidine was measured in a beta counter.

### Cytokine analysis

The levels of IL-1β, IL-6, IL-10, IL-12, IL-17, TNF and IFN-γ were determined in serum collected at day 28 using multiplex Luminex kit (Bio-Rad Laboratories, USA) according to the manufacturer’s protocol. Beads were quantified using Luminex-200 (Invitrogen) and data were analysed using MasterPlex 2010 (version 5.0.0.68).

### Statistics

All the data were analysed using Graphpad Prism (version 6.05). Differences between treated and untreated groups were compared using Mann-Whitney U test because the data did not show normal distribution. A p-value less than 0.05 was considered significant.

## Results

### Local administration of uridine inhibits development of mBSA-induced arthritis in a dose dependent manner

**Fig 1:**
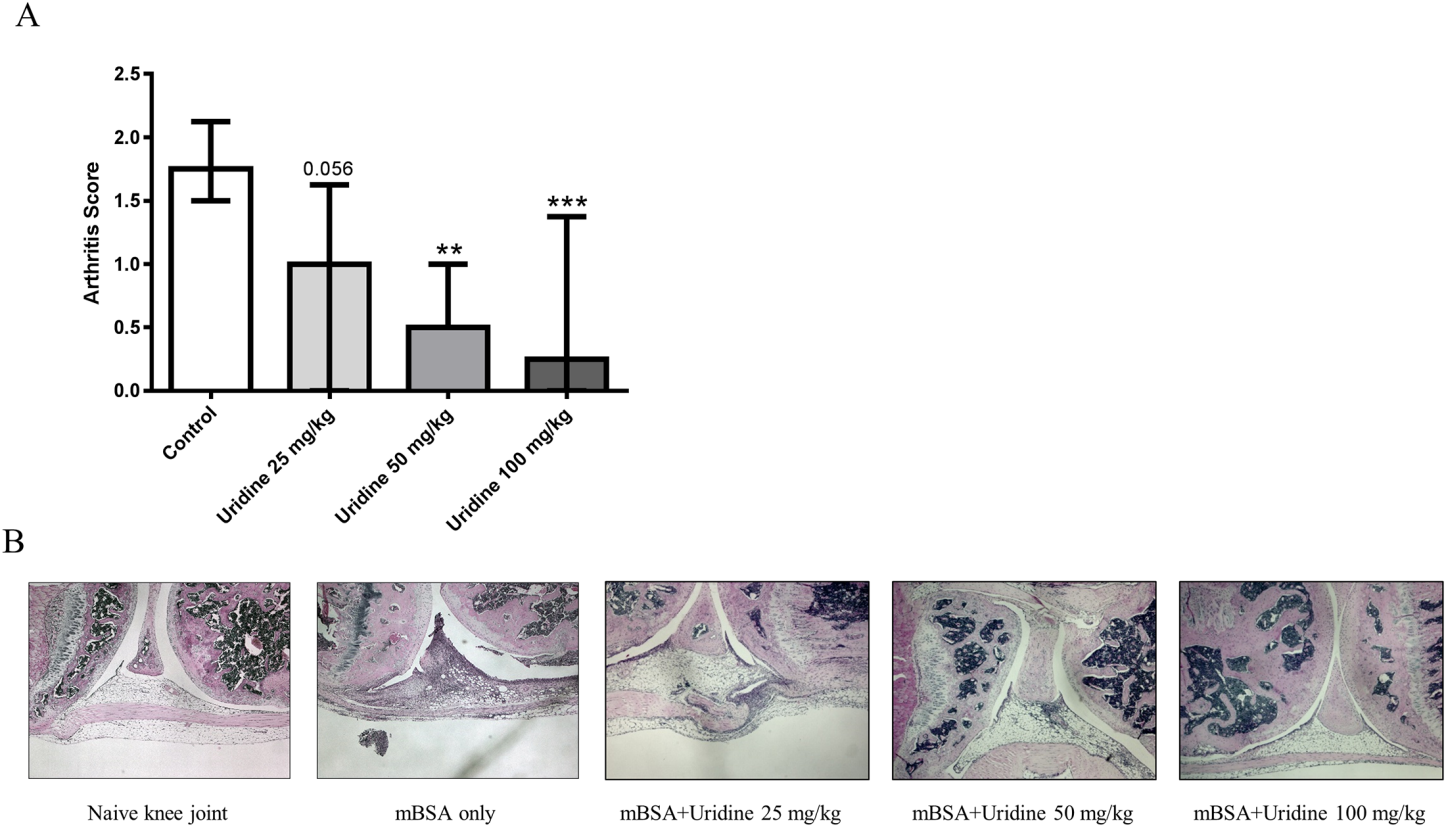
Local administration of uridine inhibits development of mBSA-induced arthritis in dose dependent manner. Single dose of 0, 25, 50 and 100 mg/kg of Uridine was co-administered locally along with 30 μg mBSA (intra-articularly) in right knee joint in mBSA-sensitized mice on day 21. At day 28, mice were sacrificed and knee joints were isolated and prepared for histopathological evaluation as described in the methods. (A) Arthritis Severity (median with interquartile range) 0 mg/kg (n = 11), 25 mg/kg (n = 6), 50 mg/kg (n = 7) and 100 mg/kg (n = 12). n>6, * p<0.05, ** p<0.01, *** p<0.001 (Mann–Whitney). Data show results of two pooled experiments. (B) Representative images of joint sections of each treatment group.

We have previously demonstrated that local administration of uridine has an anti-inflammatory effect in an asthma-like lung inflammation model [8]. To test whether uridine could inhibit development of arthritis, a single dose of 0, 25, 50, 100 mg/kg of uridine was administered in the knee joint on day 21 of AIA. Local administration of uridine led to a significant and dose dependent reduction of the arthritis score compared to control animals (Fig 1A). Mice receiving 100 mg/kg of uridine showed maximum reduction of the arthritis score compared to controls (Fig 1). Knee sections of mice that received 100 mg/kg of uridine locally were almost identical to knee sections of naive mouse knee (Fig 1B). Mice receiving 50 mg/kg and 25 mg/kg of uridine also showed a significant reduction of the arthritic score (Fig 1A) including reduced infiltration of leukocytes and hyperplasia of synovial membrane compared to controls (Fig 1B).

### Systemic administration of Uridine does not inhibit development of mBSA-induced arthritis

**Fig 2:**
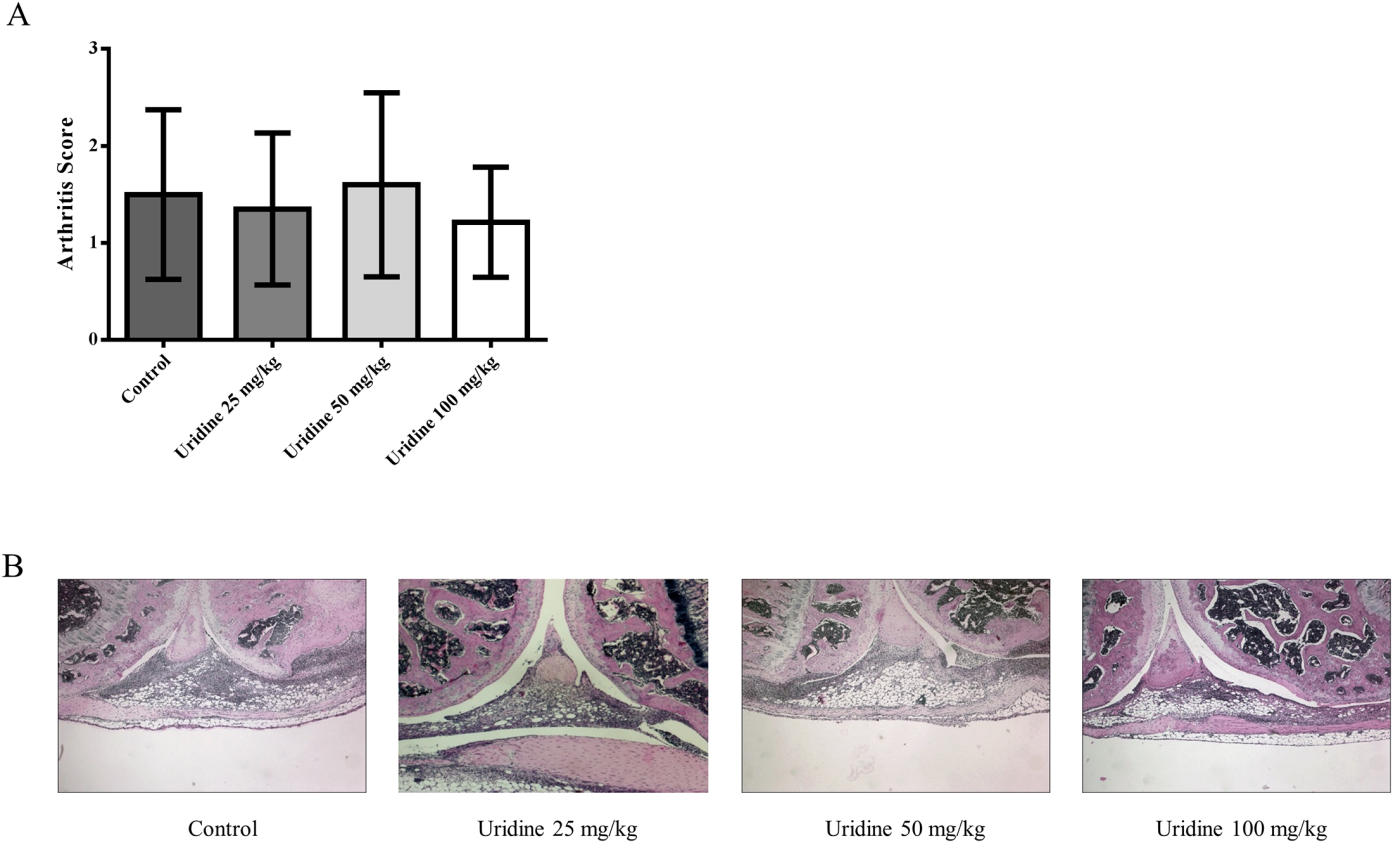
Systemic administration of uridine does not inhibit development of mBSA-induced arthritis. Multiple doses of 0, 25, 50 and 100 mg/kg of Uridine was administered systemically in mBSA-sensitized mice on day 0, 7, 14 (intra-peritoneal), 21 and 23. Arthritis was induced by injecting 30 μg of mBSA intra-articularly on day 21. At day 28, mice were sacrificed and knee joints were isolated and prepared for histopathological evaluation as described in the methods. (A) Arthritis Severity (median with interquartile range). 0 mg/kg (n = 20), 25 mg/kg (n = 10), 50 mg/kg (n = 15) and 100 mg/kg mg/kg (n = 7). Data show results of two pooled experiments. (B) Representative images of joint sections of each treatment group. * p<0.05, (Mann–Whitney).

To determine if systemic administration of uridine could have a similar anti-inflammatory effect in AIA 0–100 mg/kg uridine was injected subcutaneously (on day 0, 7, 21 and 23) and intra-peritoneally (day 14) in mice subjected to mBSA sensitization on day 1 and 7. Arthritis was induced by intra-articular injection of 30 μg of mBSA on day 21. As shown in Fig 2A, systemic administration of different doses of uridine had no effect on severity of arthritis.

We also investigated the effect of systemic administration of uridine using sustained release of uridine from slow-releasing pellets. Uridine was administered in two different doses by surgical implantation at day -1 of pellets releasing 0.75 and 1.5 mg uridine daily, respectively, during the entire 28-day period of AIA. There was no effect on AIA by sustained release of uridine (S1 Fig).

### Protection by uridine does not involve altered anti-mBSA responses in AIA

**Fig 3:**
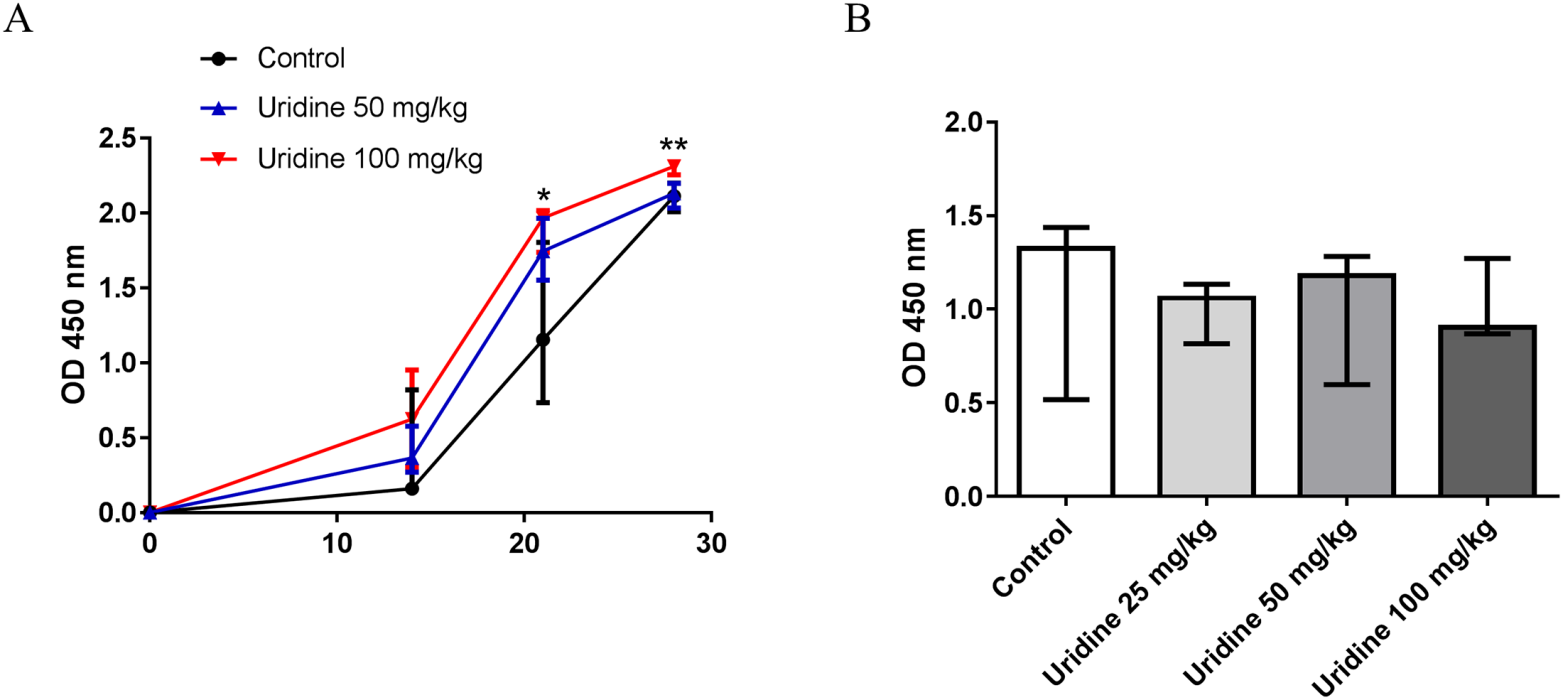
Protection by uridine does not involve altered anti-mBSA responses in AIA. Serum was collected at day 0, 14, 20 and 28 from the mBSA-sensitized mice treated systemically with multiple doses 0–100 mg/kg of uridine and at day 28 from the mBSA-sensitized mice treated locally 0–100 mg/kg with uridine and analyzed for mBSA specific total IgG using ELISA. (A) Anti-mBSA IgG serum levels in mice treated systemically with 0, 50, 100 mg/kg of uridine. (Red line-100 mg/kg n = 10, Blue line-50 mg/kg n = 7) (Black line-Control n = 7). (B) Anti-mBSA IgG serum levels in mice treated locally with 0 mg/kg (n = 6), 25 mg/kg (n = 6), 50 mg/kg (n = 7), 100 mg/kg (n = 7) of uridine. Data are expressed as median absorbance with interquartile range (450 nm). * p<0.05, ** p<0.01 (Mann–Whitney).

The protection, and lack of protection against arthritis conferred by local and systemic administration of uridine, respectively, prompted us to analyse the effects of systemic and local administration of uridine on anti-mBSA specific responses during AIA. First, we measured the levels of anti-mBSA IgG in serum on day 0, 7, 14, 20 and 28 in order to determine if systemic administration of uridine had any impact on the mBSA-specific humoral response. A minor, but still significant increase in anti-mBSA IgG was observed in animals treated with uridine, Mice receiving 100 mg/kg of uridine systemically had higher levels of serum anti-mBSA IgG on day 14, 20 and 28 than control animals receiving only vehicle (Fig 3A). A lower dose of uridine, 50 mg/kg, only resulted in increased serum levels of anti-mBSA IgG on day 20 compared to control mice. In contrast, we did not find any significant differences in serum levels of anti-mBSA specific IgG on day 28 in animals treated locally with uridine at the time of arthritis induction, and thus protected against arthritis, compared to controls (Fig 3B)

Next, we investigated if systemic administration of uridine could regulate mBSA-specific cellular responses. Lymph node cells and splenocytes isolated (day 28) from mBSA-immunized mice treated systemically with uridine or control mice were re-stimulated with mBSA (50μg/ml) *ex vivo*. There was no significant difference in leukocyte proliferation between leukocytes from uridine-treated mice and non-treated mice after re-challenge with mBSA *ex vivo* (S2 Fig).

### Protection by uridine does not involve altered cytokine profiles in AIA

**Fig 4:**
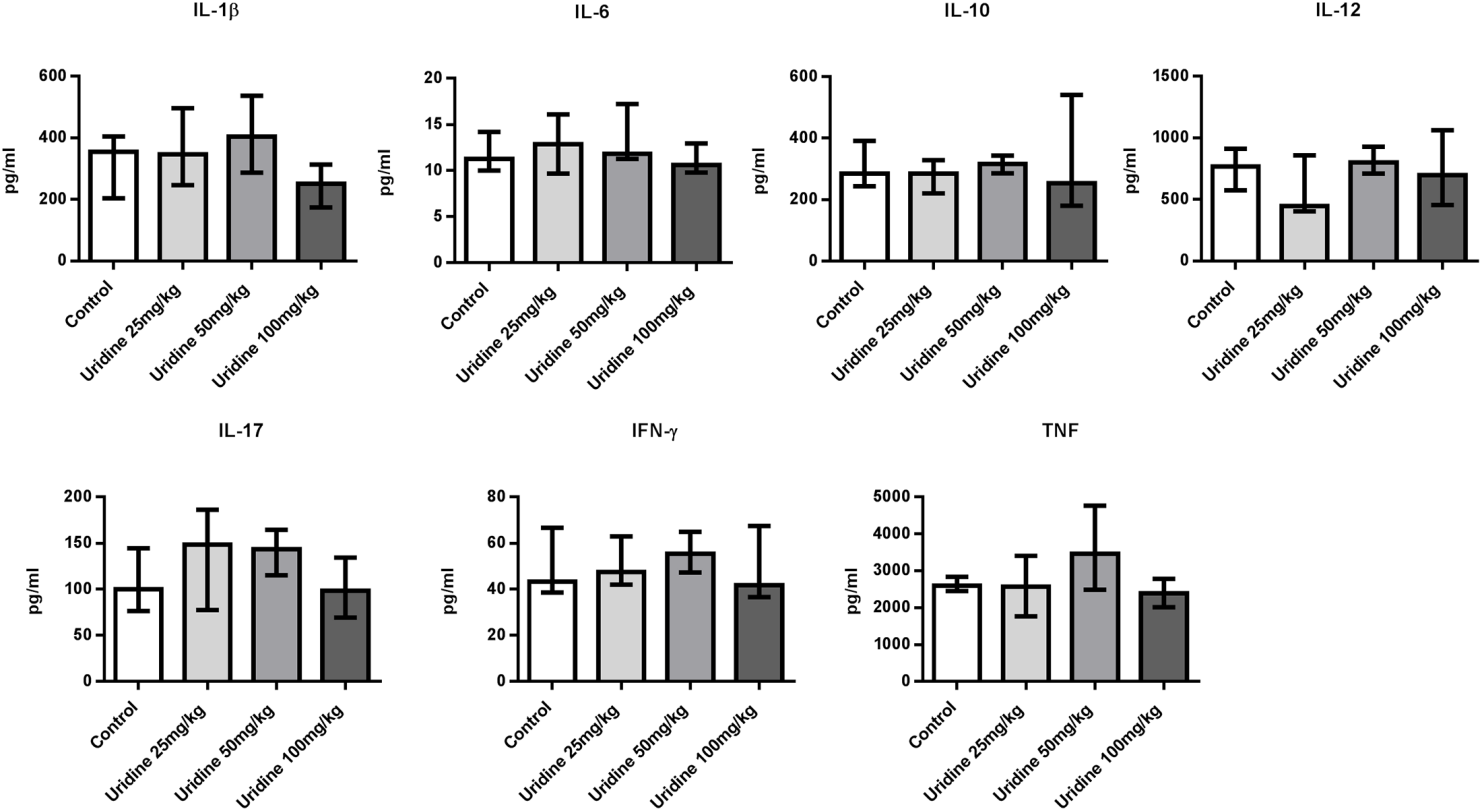
Protection by uridine does not involve altered cytokine profiles in AIA. Single dose of 0, 25, 50 and 100 mg/kg of uridine was co-administered locally along with 30 μg mBSA (intra-articularly) in right knee joint in mBSA-sensitized mice on day 21. Serum was collected at day 28 and analyzed for IL-1β, IL-6, IL-12, IL-17, TNF, IFN-γ and IL-10 levels by Luminex. Data are expressed as median pg/ml with interquartile range. 0 mg/kg (n = 11), 25 mg/kg (n = 6), 50 mg/kg (n = 7), 100 mg/kg (n = 12) of uridine.

As reported earlier, triggering of arthritis by intra-articular injection of mBSA on day 21 of AIA induces not only arthritis but also a burst of proinflammatory cytokines including IL-12, IL-17 and TNF [18]. To determine if administration of uridine had any effect on the levels of cytokines in serum we analysed the levels of IL-1β, IL-6, IL-12, IL-17, TNF, IFN-γ and IL-10 in mice subjected to systemic and local administration of uridine. Irrespective of the route of administration, we did not find any effect of uridine on serum cytokine levels on day 28 of AIA (Fig 4 and S3 Fig), with the exception of a minor, but still significant reduction in serum TNF levels in mice systemically treated with uridine (100 mg/kg) (S3 Fig).

### Local administration of uridine suppressed synovial expression of TNF and IL-6

**Fig 5:**
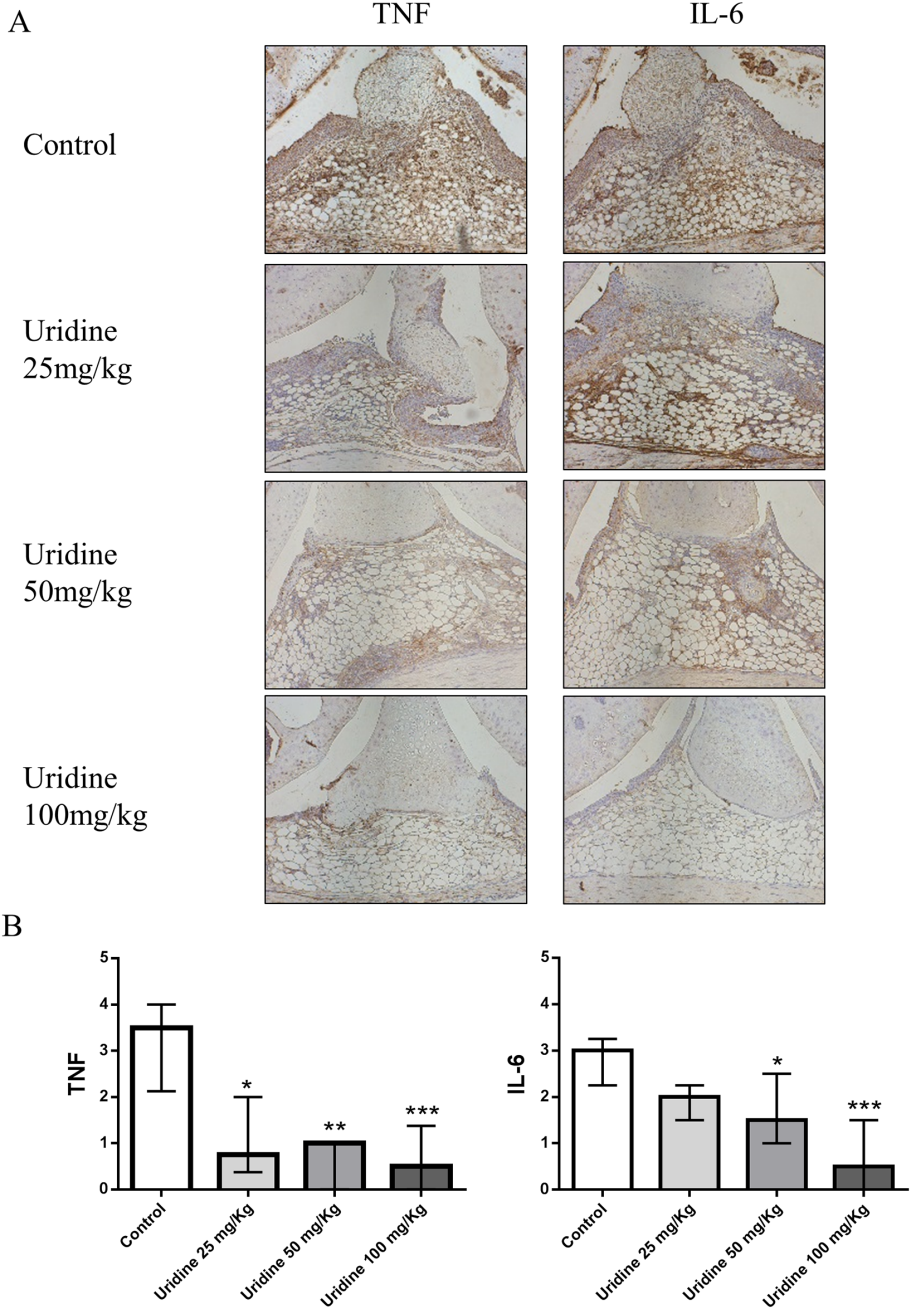
Local administration of uridine suppressed synovial expression of TNF-α and IL-6. Single dose of 0, 25, 50 and 100 mg/kg of uridine was co-administered locally along with 30 μg mBSA (intra-articularly) in right knee joint in pre-sensitized mice on day 21. At day 28, mice were sacrificed and knee joints were isolated and prepared for immunohistochemistry staining and evaluation. (A) Representative Immunohistochemistry images of non-treated and treated groups for TNF-α and IL-6 expression. (B) Immunohistochemistry scoring of non-treated and treated groups for TNF-α and IL-6 expression (median with interquartile range). 0 mg/kg (n = 6), 25 mg/kg (n = 6), 50 mg/kg (n = 7), 100 mg/kg (n = 12) of uridine. * p<0.05, ** p<0.01, *** p<0.001(Mann–Whitney).

To further understand the mechanism behind the protective effect of local administration of uridine in AIA, we used immunohistochemistry to determine the effect of uridine on synovial expression of TNF and IL-6. There was a clear and significant reduction of synovial expression of TNF in uridine treated animals compared to controls (Fig 5). Mice receiving 100 mg/kg of uridine showed maximum suppression of synovial expression of TNF compared to controls (77%). Interestingly, a remarkable and significant (68%) reduction of TNF expression was found already at the lowest dose of uridine (25 mg/kg). A significant dose dependent response was observed in synovial expression of IL-6 in mice treated locally with uridine. In a similar vein, maximum reduction of synovial IL-6 expression was attained in mice that received 100 mg/kg (71%) as seen in Fig 5B, whereas the lowest dose (25 mg/kg), did not result in significant reduction of IL-6 expression.

### Local administration of uridine suppressed synovial cell influx

**Fig 6:**
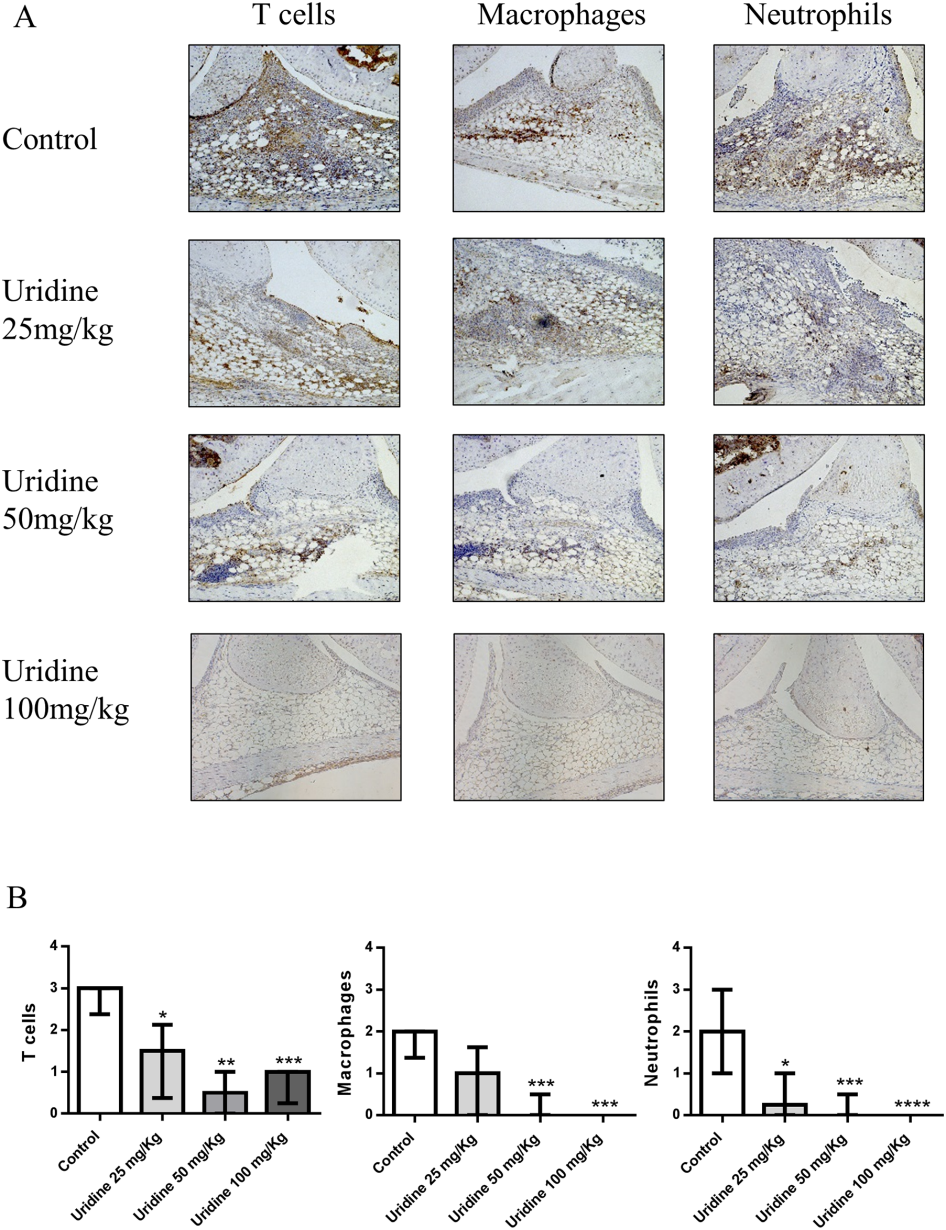
Local administration of uridine suppressed synovial cell influx. Single dose of 0, 25, 50 and 100 mg/kg of uridine was co-administered locally along with 30 μg mBSA (intra-articularly) in right knee joint in pre-sensitized mice on day 21. At day 28, mice were sacrificed and knee joints were isolated and prepared for immunohistochemistry staining and evaluation. (A) Representative Immunohistochemistry images of non-treated and treated groups for T cells, Macrophages and Neutrophils. (B) Immunohistochemistry scoring of non-treated and treated groups for T cells, Macrophages and Neutrophils (median with interquartile range). 0 mg/kg (n = 6), 25 mg/kg (n = 6), 50 mg/kg (n = 7), 100 mg/kg (n = 12) of uridine. * p<0.05, ** p<0.01, *** p<0.001, **** p<0.0001 (Mann–Whitney).

Next, we investigated the effect of locally administered uridine on the influx of T cells, macrophages and neutrophils into the synovium in AIA. Local administration of uridine significantly decreased the influx of T cells, macrophages and neutrophils in a dose dependent manner as shown in Fig 6A and 6B. Mice that received the highest dose of uridine (100 mg/kg) had a 67% reduction T cells, 95% reduction in macrophages and a 98% reduction in neutrophils. Thus, the highest dose of 100 mg/kg almost abrogated neutrophil and macrophage influx into the synovium. Also, the lower doses of uridine, 25 and 50 mg/kg significantly reduced the influx of T cells and neutrophils, whereas only the 100 mg and 50 mg doses significantly reduced influx of macrophages. A lesser (67%) reduction in T cell influx was observed compared to macrophages and neutrophils (95 and 98% respectively). However, the absolute reduction in T cell infiltration was higher as T cells constitute more of the synovial infiltrate than macrophages and neutrophils. (Fig 6).

### Local administration of uridine supressed synovial expression of ICAM-1 and CD18

**Fig 7:**
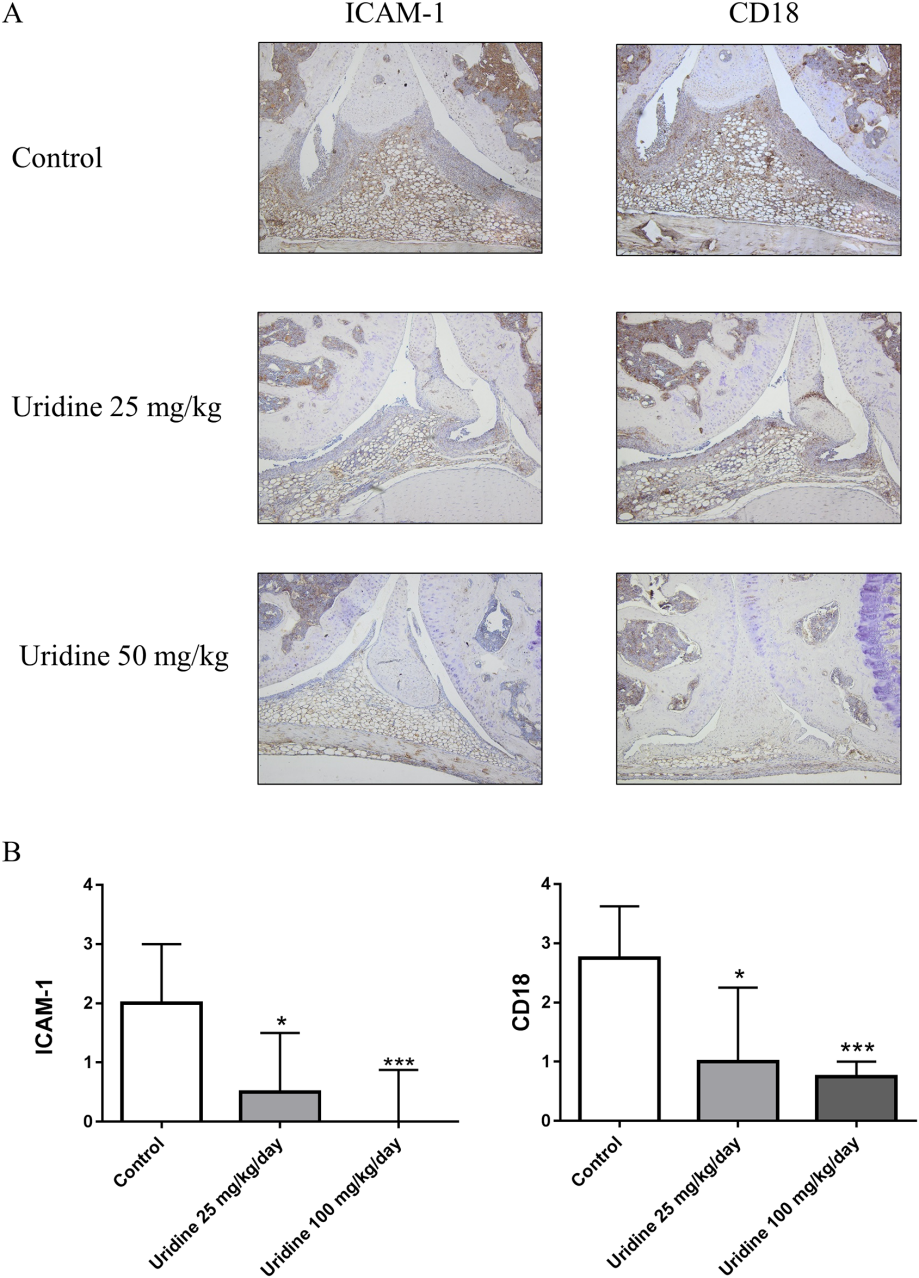
Local administration of uridine supressed synovial expression of ICAM-1 and CD18. Single dose of 0, 25, 50 and 100 mg/kg of uridine was co-administered locally along with 30 μg mBSA (intra-articularly) in right knee joint in pre-sensitized mice on day 21. At day 28, mice were sacrificed and knee joints were isolated and prepared for immunohistochemistry staining and evaluation. (A) Representative Immunohistochemistry images of non-treated and treated groups for ICAM-1 and CD18 expression. (B) Immunohistochemistry scoring of non-treated and treated groups for ICAM-1 and CD18 expression (median with interquartile range). 0 mg/kg (n = 6), 25 mg/kg (n = 6), 100 mg/kg (n = 12) of uridine. * p<0.05, ** p<0.01, *** p<0.001 (Mann–Whitney).

Recruitment of leukocytes to the site of injury is one of the early events in inflammation. This involves expression and activation of various cell and endothelial adhesion molecules. Supressed synovial cell influx in uridine treated joint motivated us to investigate the effects of local administration of uridine on synovial ICAM-1 and CD18. There was a significant suppression of ICAM-1 and CD18 in uridine treated mice than the controls. In mice receiving 100mg/kg of uridine, there was a 85% and 73% reduction of synovial expression of ICAM-1 and CD18 respectively (Fig 7A and 7B). A lower but significant reduction of ICAM-1 (67%) and CD18 (50%) expression was noticed with 25 mg/kg of uridine (Fig 7A and 7B).

## Discussion

Rheumatoid arthritis is a chronic inflammatory disease with a strong autoimmune component. The pathogenesis of rheumatoid and experimental arthritis involves migration of leukocytes from blood vessels into synovial tissues. Various inflammatory mediators released by the leukocytes are partly responsible for the persistent inflammation, chronic recruitment of effector cells and erosion of articular cartilage associated with RA.

Since recruitment of leukocytes is probably the most critical step for mounting an adequate inflammatory response, blocking leukocyte extravasation is an interesting target for therapeutic modulation of inflammation.

We have previously demonstrated that intra-tracheal administration of uridine has a clear and significant anti-inflammatory effect in a sephadex-induced lung inflammation model [8]. In the present study, we show that uridine exhibits an anti-inflammatory effect also in arthritis.

Uridine is known to inhibit leukocyte adhesion to human endothelial cells in vitro [19], which may thus inhibit extravasation. Here we have shown for the first time that uridine suppressed both ICAM-1 and CD18 expression in the synovium (Fig 7B). We report that uridine clearly inhibited extravasation to the synovium, including cells producing TNF and IL-6, whereas TNF and IL-6 in serum was equally high as in arthritic, non-treated controls. We, therefore postulate that uridine's protective effect in AIA is primarily mediated through inhibition of adhesion molecule expression and secondarily through arrested TNF and IL-6 production in situ as a function of reduced inflammatory cell extravasation. However, a specific inhibitory effect on the production of certain cytokines cannot be entirely ruled out. Since uridine in high doses almost abrogated neutrophil and macrophage influx (95 and 98% respectively) into the synovium, it is clear that uridine has a pronounced effect on the acute phase of the inflammatory reaction. Taking into consideration that T cells constitute more of the synovial infiltrate than macrophages and neutrophils, respectively, the reduction of T-cell influx observed (67%) suggests a promising effect on T cells as well.

Although local administration of uridine did not affect serum cytokine levels in AIA, intra-tracheal administration of uridine has been shown to inhibit the release of TNF, IL-6, IL-8 into bronchoalveolar fluid [8, 9]. Further, treatment of A549 cells with uridine inhibits IL-4 induced production of IL-6 and IL-8. Thus an effect on local cytokine production in AIA by uridine may not be completely ruled out and could be supported by our finding that systemic administration of uridine caused a small, but significant reduction of TNF levels in serum (S3 Fig). Apart from inhibiting TNF production, uridine inhibited extravasation via reduction of ICAM-1 and CD18 (LFA-1), which facilitate extravasation and are regulated by TNF [2, 20]. Finally, uridine clearly reduces the number of TNF and IL-6 producing cells in the inflamed joint, which could also speak for an inhibiting role on cytokine production, although this effect cannot be separated from inhibited extravasation.

As local administration of uridine was efficacious, we continued to investigate if systemic administration of uridine was equally efficacious in protecting against AIA. Systemic administration by subcutaneous or intraperitoneal injection of multiple doses (day 0, 7, 14, 21 and 23) of uridine (25–100 mg/kg) had no effect on AIA. Further, experiments with surgically implanted pellets, as an alternative mode of systemic delivery did not have any demonstrable effect on arthritis severity either. Exogenously administered uridine is rapidly metabolized by the liver [21] and has a half-life of less than 2 minutes [22]. The rapid pharmacokinetics of uridine and difficulties associated with achieving significant levels of any drug locally in the knee joint could possibly explain the lack of effect observed upon systemic administration.

In an effort to delineate the mechanism behind uridine-mediated protection against OVA-induced lung inflammation, Müller et al induced lung inflammation by intra-tracheal transfer of OVA-pulsed DC followed by intra-tracheal challenge with OVA. In line with the data presented here on AIA, direct administration of uridine at the site of antigen re-challenge (trachea) in pre-sensitized animals clearly prevented antigen-induced inflammation. In contrast, if uridine was administered by pre-treating the OVA-DC with uridine before transfer, a strong inflammatory reaction still ensued [9]. This observation is consistent with our observation here that only direct, local administration was effective and suggests that uridine may act directly on the vessel wall preventing extravasation of leukocytes to the site of antigen re-challenge.

Antigen-specific T cell and antigen-specific humoral responses are important factors influencing disease progression in AIA. We found no effect on anti-mBSA IgG in animals protected by local administration of uridine compared to controls. A small increase in serum levels of anti-mBSA IgG were found upon systemic administration of uridine (that did not protect against AIA). Thus, protection against AIA by local uridine administration does not involve modulation of the systemic anti-mBSA response and further underscores our postulation that the principal effect of uridine here is direct and local.

TNF, IL-6, IL-1β are pro-inflammatory components involved in the pathogenesis of both RA and AIA [3, 23] and high levels of these cytokines in serum and synovium are a hallmark of active disease [24]. Likewise, the intra-articular injection of mBSA that triggers arthritis in AIA, is accompanied by a strong recall response of proinflammatory cytokines in serum, including IL-6, IL-10, IL-12, and TNF [18]. In this model of arthritis, the levels of these proinflammatory cytokines clearly rise after the booster immunization and decline within days thereafter. In the absence of intra-articular injection of mBSA the levels remain low, whereas if arthritis is triggered by intra-articular injection of mBSA, the serum levels of IL-6, IL-12, IL-17, IFN-γ and TNF increase rapidly again [18]. Intriguingly, although intra-articular delivery of uridine clearly protected against arthritis, it did not prevent this recall response as serum levels of pro-inflammatory cytokines were equally (IL-1β, IL-6, IL-12, IL-17, TNF and IFN-γ) high in uridine treated and control animals one week after intra-articular injection of mBSA (Fig 4A). This observation further strengthens the view that local uridine administration directly prevents inflammation without affecting cellular antigen specific responses.

The purinogenic P2Y G-protein coupled receptors (P2Y2, P2Y4 and P2Y6) could theoretically mediate the effects of uridine indirectly after conversion of uridine by kinases to UDP and UTP [25]. The effect of purinogenic receptor signalling on inflammation however remains controversial with both pro and anti-inflammatory effects being described in the literature [26], [27]. As mentioned earlier, intra-tracheal administration of uridine suppresses OVA-induced lung inflammation [9] but does so also in the absence of a functional P2Y6R (Marco Idzko, University of Freiburg, personal communication). Thus, because uridine can exert its anti-inflammatory effect also in the absence of a P2Y receptor we believe that the anti-inflammatory property uridine does not require conversion into phosphorylated derivatives thereof (UDP and UTP).

In conclusion, the present study demonstrates a novel anti-inflammatory property of uridine in antigen-induced arthritis. Inflammation is blocked effectively upon local administration of uridine, but without affecting general systemic immune parameters. This makes uridine a suitable new anti-rheumatic therapeutic candidate for further evaluation.

## Supporting Information

S1 Fig Sustained, systemic administration of uridine has no effect on mBSA-induced arthritis. Sustained release pellets (placebo, uridine 0.75 mg/day and 1.5 mg/day) were surgically implanted in the mice before the first immunization during AIA. Arthritis was induced by injecting 30 μg of mBSA intra-articularly on day 21. At day 28, mice were sacrificed and knee joints were isolated and prepared for histopathological evaluation. Arthritis Severity (median with interquartile range). Placebo (n = 6), uridine 0.75 mg/day (n = 9), uridine 1.5 mg/day (n = 9).
(TIF)

S2 Fig Systemic administration of uridine has no effect on mBSA-induced proliferation *ex-vivo*. At day 28, single cell suspensions were prepared separately from draining lymph nodes and spleens isolated from mBSA-senstitized mice as described in methods section. (A) lymph nodal cells and (B) splenocytes were restimulated with 50 μg/ml of mBSA for 48 hours. ^3^H-thymidine was added and cells were incubated for an additional 20 hours. Cells harvested and the amount of incorporated tritiated thymidine was measured in a beta counter. Values expressed as cpm (median with interquartile range). 0 mg/kg (n = 10), 25 mg/kg (n = 10), 50 mg/kg (n = 9).
(TIF)

S3 Fig Systemic administration of uridine has no effect on cytokine profile in AIA. Multiple doses of 0–100 mg/kg of uridine was administered systemically in mBSA-sensitized mice on day 0, 7, 14 (intra-peritoneal), 21 and 23. Arthritis was induced by injecting 30 μg of mBSA intra-articularly on day 21. Serum was collected at day 28 and analyzed for IL-1β, IL-6, IL-12, IL-17, TNF and IFN-γ and IL-10 levels by Luminex. Data are expressed as median pg/ml with interquartile range. 0 mg/kg (n = 5), 100 mg/kg (n = 4) of uridine, * p<0.05, (Mann–Whitney).
(TIF)
