# Supplementary figures and images for: Local but Not Systemic Administration of Uridine Prevents Development of Antigen-Induced Arthritis
Source: PLoS One. 2015 Oct 29;10(10):e0141863. doi: 10.1371/journal.pone.0141863 (PMC4625961; doi:10.1371/journal.pone.0141863)

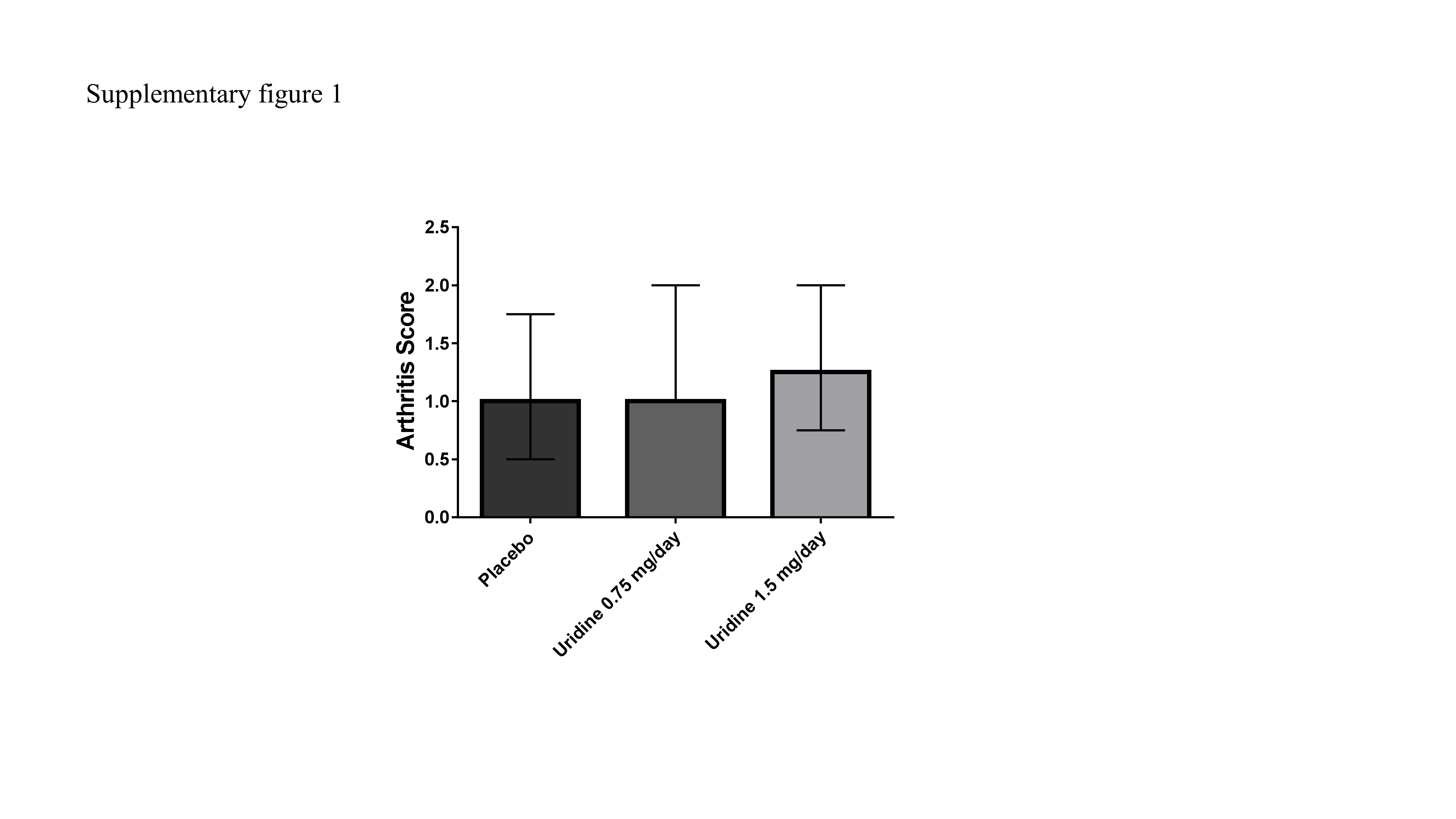

Supplement: S1 Fig — Sustained release pellets (placebo, uridine 0.75 mg/day and 1.5 mg/day) were surgically implanted in the mice before the first immunization during AIA. Arthritis was induced by injecting 30 μg of mBSA intra-articularly on day 21. At day 28, mice were sacrificed and knee joints were isolated and prepared for histopathological evaluation. Arthritis Severity (median with interquartile range). Placebo (n = 6), uridine 0.75 mg/day (n = 9), uridine 1.5 mg/day (n = 9). (TIF) [file pone.0141863.s001.tif]

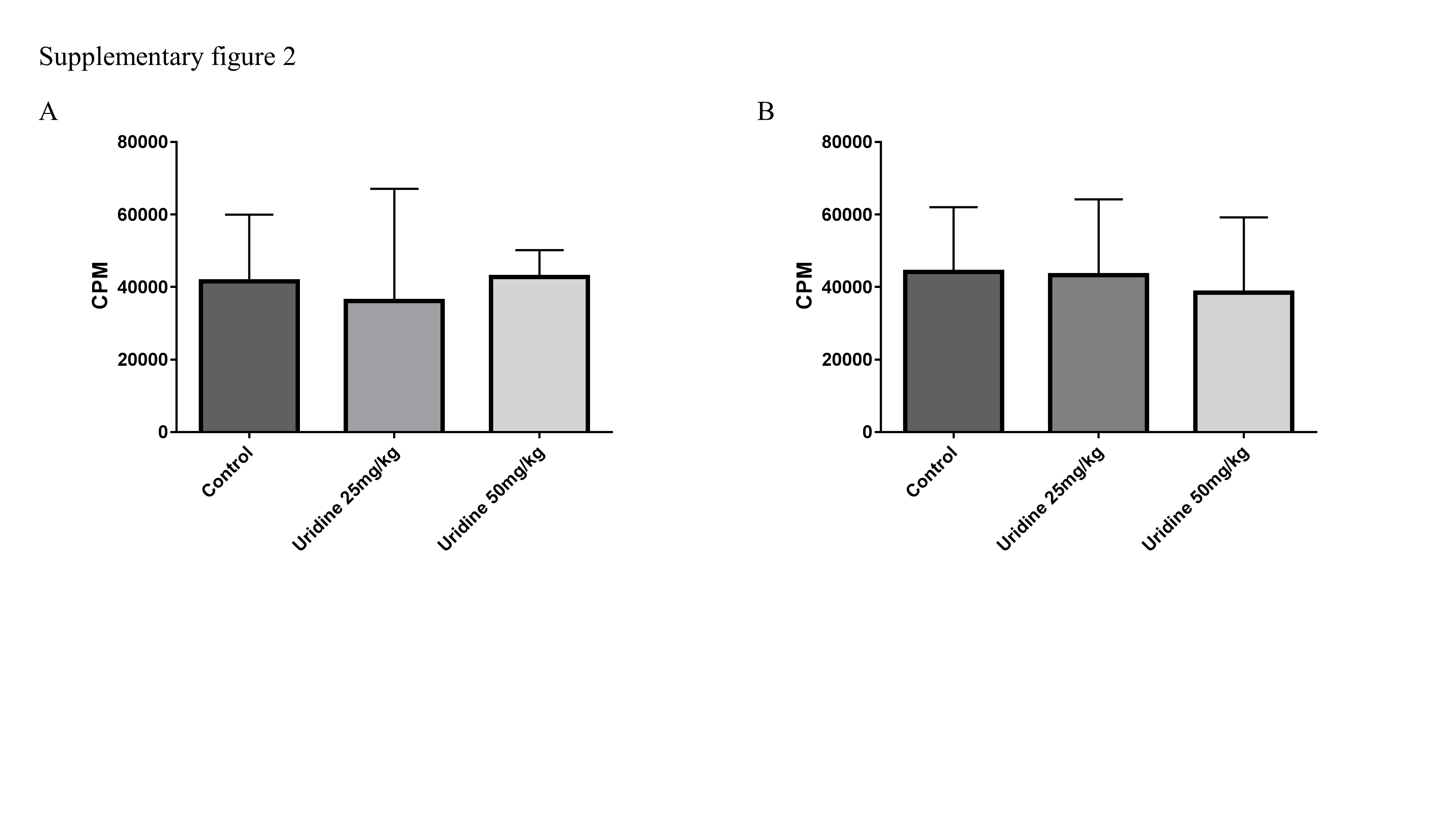

Supplement: S2 Fig — At day 28, single cell suspensions were prepared separately from draining lymph nodes and spleens isolated from mBSA-senstitized mice as described in methods section. (A) lymph nodal cells and (B) splenocytes were restimulated with 50 μg/ml of mBSA for 48 hours. 3H-thymidine was added and cells were incubated for an additional 20 hours. Cells harvested and the amount of incorporated tritiated thymidine was measured in a beta counter. Values expressed as cpm (median with interquartile range). 0 mg/kg (n = 10), 25 mg/kg (n = 10), 50 mg/kg (n = 9). (TIF) [file pone.0141863.s002.tif]

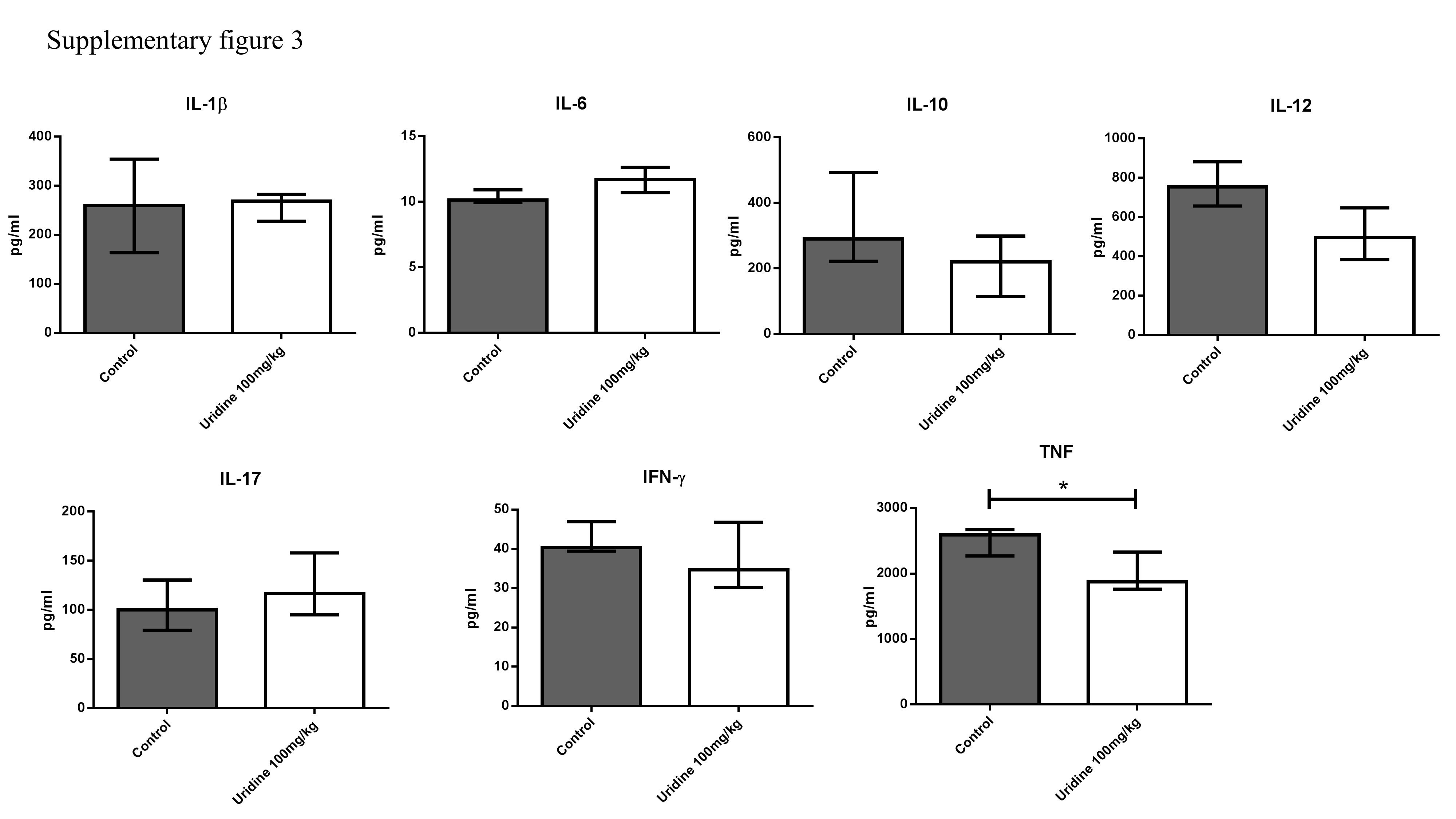

Supplement: S3 Fig — Multiple doses of 0–100 mg/kg of uridine was administered systemically in mBSA-sensitized mice on day 0, 7, 14 (intra-peritoneal), 21 and 23. Arthritis was induced by injecting 30 μg of mBSA intra-articularly on day 21. Serum was collected at day 28 and analyzed for IL-1β, IL-6, IL-12, IL-17, TNF and IFN-γ and IL-10 levels by Luminex. Data are expressed as median pg/ml with interquartile range. 0 mg/kg (n = 5), 100 mg/kg (n = 4) of uridine, * p<0.05, (Mann–Whitney). (TIF) [file pone.0141863.s003.tif]
